# Relationships of sleep disturbance, intestinal microbiota, and postoperative pain in breast cancer patients: a prospective observational study

**DOI:** 10.1007/s11325-020-02246-3

**Published:** 2020-11-19

**Authors:** Zhi-Wen Yao, Bing-Cheng Zhao, Xiao Yang, Shao-Hui Lei, Yu-Mei Jiang, Ke-Xuan Liu

**Affiliations:** Department of Anesthesiology, Nanfang Hospital, Southern Medical University, Guangzhou, Guangdong People’s Republic of China

**Keywords:** Preoperative sleep disturbance, Intestinal microbiota, Acute postoperative pain

## Abstract

**Purpose:**

Our study was designed to examine the possible relationship between gut microbiota, sleep disturbances, and acute postoperative pain.

**Methods:**

Using 16S rRNA sequencing, we analyzed preoperative fecal samples from women undergoing breast cancer surgery. Preoperative sleep disturbance was evaluated with the Pittsburgh Sleep Quality Index (PSQI) questionnaire. Peak and average pain at rest and movement were evaluated 24 h after surgery, using a numerical rating scale (NRS). Preoperative symptoms of depression and anxiety were assessed with the Patient Health Questionnaire-9 (PHQ-9) and Generalized Anxiety Disorder-7 (GAD-7), respectively. Inflammation was measured using white blood cell and neutrophil counts, together with platelet-lymphocyte ratio, and neutrophil-lymphocyte ratio.

**Results:**

Preoperative sleep disturbance was associated with more severe acute postoperative pain. At the phylum level, women with poor sleep quality had higher relative abundance of Firmicutes (*p* = 0.021) and lower relative abundance of Bacteroidetes (*p* = 0.013). At the genus level, women with poor sleep quality harbored higher relative abundance of Acidaminococcus and lower relative abundance of several genera. The genus Alloprevotella was negatively associated with peak pain at movement during the first 24 h (*r* = − 0.592, *p* < 0.001). The genus Desulfovibrio was negatively associated with symptoms of anxiety (*r* = − 0.448, *p* = 0.006). However, partial correlations suggested that the relationship between Alloprevotella and peak pain at movement during the first 24 h was not statistically significant after controlling for sleep (*r* = − 0.134, *p* = 0.443).

**Conclusion:**

These findings suggest that the changed gut microbiota may be involved in sleep-pain interaction and could be applied as a potential preventive method for postoperative pain.

**Trial registration:**

The present clinical study has been registered on Chinese Clinical Trial Registry (www.chictr.org.cn); the clinical trial registration number is ChiCTR1900021730; the date of registration is March 7, 2019.

**Supplementary Information:**

The online version contains supplementary material available at 10.1007/s11325-020-02246-3.

## Introduction

Breast cancer is the most frequently diagnosed cancer in women and the second leading cause of cancer-related deaths among women worldwide [1]. Sleep disturbance is a common and significant symptom in women who undergo treatment for breast cancer [2, 3]. The incidence of sleep disturbance prior to surgery ranges from 33 to 88% and is associated with cancer incidence [4–7]. Pain after breast surgery is another common problem in women treated for breast cancer, which can have persisting negative consequences, such as exacerbation of some side effects (in particular, nausea, and fatigue), delayed recovery, development of chronic persistent pain in the surgical area, mental stress, and lower quality of life [8]. A relationship between sleep disturbance and pain has long been recognized. However, it is difficult to separate the effects of sleep disturbance on pain from the effects of pain on sleep disturbance. Preliminary research with healthy participants has shown that disturbed sleep can induce generalized hyperalgesia [9, 10], supporting the hypothesis regarding interactions between sleep disturbance and acute pain. In addition, other emerging evidence suggests that poor sleep before surgery is associated with more severe postoperative pain [11, 12].

The collective genome of the human gut microbiota, which is composed of 10^13^ to 10^14^ microorganisms, includes 2.8 billion base pairs, equivalent to at least 100 times as many genes as the human genome [13]. The gut microbiota has formed a close relationship with its host over the course of long-term evolution and is currently considered one of the most important factors involved in the bidirectional interaction of the gut-brain axis [14, 15]. It has been suggested that the composition of the gut microbiota can be altered by partial sleep deprivation in humans and chronic sleep disruption in mice [16, 17]. Furthermore, studies in germ-free mice and animals treated with antibiotics have indicated that the gut microbiota may participate in the pain perception processes [18, 19]. However, whether or not the gut microbiota is involved in the sleep-pain interaction remains unclear. In this preliminary study, we examined the possible association between the gut microbiota composition, preoperative sleep disturbance, and postoperative pain. Specifically, we examined whether the gut microbiota played a role in sleep-pain interaction in a sample of patients with breast cancer undergoing elective surgery.

## Materials and methods

### Participant information and procedures

This prospective, observational, clinical study was approved by the Medical Ethics Committee of Nanfang Hospital (NFEC-2019-001), Southern Medical University (Guangdong, China). After receiving a written description of the aims of this study, participants planning to undergo a surgical procedure for breast cancer, recruited from Nanfang Hospital from March 10, 2019, to May 25, 2019, provided written informed consent to participate in the study. The demographic, clinicopathological, and histopathology data of patients were collected from the hospital’s electronic medical records. Nutritional and pain experience data were collected using interview-based questionnaires, which were administered following screening for eligibility conducted at the start of the interview (see Additional file 1).

All patients had undergone a core needle biopsy before study enrollment and the diagnosis of cancer was made according to the criteria of the 2018 National Comprehensive Cancer Network Breast Cancer Version 3 [20]. Patients were included in this study if they were about to undergo surgery under the standardized anesthesia protocol (see Additional file 2); a decision made to minimize variability due to different doses of analgesics or types of anesthesia. Patients were asked to provide a fecal sample 1–3 days before surgery. On the day when the fecal sample was collected, interviews concerning preoperative sleep disturbance, depression, and anxiety were conducted by a researcher purposely trained for the study. Acute postoperative pain was evaluated 24 h after surgery during a face-to-face interview with an anesthesiologist on our team.

### Measures

#### Sleep quality

The Pittsburgh Sleep Quality Index (PSQI) questionnaire is a standardized, self-administered questionnaire to assess subjective sleep quality over a 1-month period. A score ≥ 6 indicates poor sleep [21]. In cancer cohorts, PSQI has been reported as a reliable and accurate assessment tool [22].

#### Acute postoperative pain

Acute postoperative pain was chosen as an outcome of interest, as it has been reported as a significant predictor of recovery and survival in breast cancer patients [23]. Patients rated their acute postoperative pain on a 0–10 numerical rating scale (NRS; 0 indicating no pain, 10 indicating worst pain imaginable) at rest and movement during 24 h after surgery [24]. Peak pain at movement 24 h after surgery was selected as primary outcome in this study as it tended to reach its peak severity at that point. A score ≥ 4 was considered clinically meaningful acute pain [25], based on a previously established cutoff associated with a significant change to physical and emotional functioning [26].

#### Demographic and clinical variables

Information on age, body mass index (BMI), education, marital status, and presence or absence of preoperative breast pain was recorded during preoperative interviews. One patient had undergone a breast-conserving surgery (3%), while the majority had undergone a mastectomy with (81%) or without axillary lymph node dissection (11%). The intercostobrachial nerve, which is at risk of damage in mastectomy and can result in chronic neuropathic pain, was not specifically preserved during the surgery [27]. Serum inflammatory markers examined by routine blood test were white blood cell count, neutrophils count, platelet-lymphocyte ratio, and neutrophil-lymphocyte ratio [28].

#### Emotional functioning

General emotional distress and disease-specific emotional functioning were assessed before surgery using two self-administered questionnaires [29]. The Patient Health Questionnaire-9 (PHQ-9) [30], which is a tool with well-established reliability and validity for depression screening in cancer patients, was used with the recommended cutoff score of ≥ 8 considered indicative of depressive symptoms [31]. The Generalized Anxiety Disorder Screener-7 (GAD-7) [32], a questionnaire with established reliability and constructive validity to assess the present degree of anxiety in cancer patients, was used with the recommended cutoff score of ≥ 7 considered to indicate symptoms of anxiety [33].

#### Fecal sample collection and sequencing

Fecal samples were self-collected by the participants at 6:30–8:30 am, 1–3 days before surgery. Each sample was then submerged in a tube with RNAlater (Invitrogen, Vilnius, Lithuania). The volume ratio of RNAlater to the sample was 1:5–10 [34]. Fecal samples were then delivered to the laboratory facilities within 6 h. After arrival at the lab, each sample was thoroughly mixed in an electronic oscillator and subsequently divided into 3 aliquots and stored at − 80 °C before sequencing. RNAlater was removed after centrifugation. Genomic DNA from fecal samples was extracted with a modification of the stool QIAamp DNA Stool mini kit (QIAGEN, Valencia, CA). Extracted DNA samples were amplified, DNA libraries were constructed, and sequencing was performed using the Illumina HiSeq (Guangzhou Gene Denovo Co. Ltd., Guangzhou, China). The details of the procedure are available in an online supplement (see Additional file 3).

### Statistical analyses

The primary outcome was peak pain score upon movement (NRS 0–10) during the first 24 h after surgery, with NRS ≥ 4 considered meaningful acute pain. Participants were divided into two groups based on their preoperative PSQI score with a cutoff of 6, good sleep quality was a PSQI < 6 and poor sleep quality was with a PSQI ≥ 6. PSQI and NRS results were presented as means ± standard deviation, unless otherwise indicated. Descriptive data were presented as means ± standard deviation or percentages. Continuous variables were compared using the Mann-Whitney rank test or a Student's *t* test, depending on distribution of the data. Categorical variables were compared using a two-tailed Fisher’s exact test. The associations between postoperative pain and age, BMI, education, marital status, preoperative pain in the affected breast, and preoperative symptoms of anxiety, and depression were examined using Spearman correlation. Partial correlations were used to determine whether the association between gut microbiota composition and the NRS performance was independent of sleep disturbance. Strain composition analysis, alpha diversity analysis, beta diversity, and function analysis were performed using QIIME (1.9.1) [35]. Statistical analyses were performed using IBM SPSS Statistics version 21.0 (IBM Corp., Armonk, NY, USA.).

## Results

Two participants withdrew from the study after enrollment for personal reasons; the final study participants included 36 patients. None of the patients reported taking any kind of medication to improve sleep quality or reduce anxiety and depressive symptoms during the study period.

### Sleep quality and demographic characteristics

The incidence of preoperative sleep disturbance was 53%. Women were divided into two groups according to their preoperative PSQI score as mentioned above, one group with sleep disturbance (SD) and the other group without sleep disturbance (nSD). The mean PSQI score of SD group was 10.7 ± 1.8, while that of nSD group was 2.9 ± 0.9 (*p* < 0.001). There was no difference in demographic characteristics (age, BMI, education, marital status, preoperative pain in the affected breast, and serum inflammatory markers including white blood cell count, neutrophils count, platelet-lymphocyte ratio, and neutrophil-lymphocyte ratio) between the two groups except for preoperative symptoms of depression and anxiety, which was more obvious in SD group (*p* < 0.001). The number of patients with college education in the sleep disturbance group was higher than that of nSD group (*p* = 0.09) (Table 1).

**Table 1:** Demographic characteristics, sleep quality, and acute postoperative pain

	Women with sleep disturbance	Women without sleep disturbance	*p* value
Age (years) (mean ± SD)	46.95 ± 9.87	49.18 ± 5.82	0.41
BMI (kg/m^2^) (mean ± SD)	23.37 ± 3.34	24.80 ± 2.86	0.18
College education, *n* (%)	7 (36.8%)	2 (11.8%)	0.09
Married, *n* (%)	17 (89.5%)	15 (88.2%)	0.67
Presence of preoperative breast pain, *n* (%)	2 (10.5%)	3 (17.6%)	0.45
PSQI score (mean ± SD)	10.68 ± 1.83	2.94 ± 0.90	< 0.001^**^
PHQ-9 score (mean ± SD)	6.11 ± 5.26	1.65 ± 2.34	< 0.001^**^
GAD-7 score (mean ± SD)	5.32 ± 5.07	2.17 ± 2.19	0.03^*^
White blood cell count (mean ± SD)	6.19 ± 2.11	5.94 ± 1.79	0.70
Neutrophils count (mean ± SD)	3.85 ± 1.69	3.60 ± 1.44	0.64
Platelet-lymphocyte ratio (mean ± SD)	166.11 ± 92.67	160.26 ± 48.30	0.82
Neutrophil-lymphocyte ratio (mean ± SD)	2.22 ± 1.31	2.02 ± 0.85	0.59
Ask for additional analgesia, *n* (%)	9 (47.4%)	2 (11.8%)	0.03^#^

*Abbreviations*: *SD*, standard deviation; *BMI*, body mass index; *n*, sample size; *n*, sample size; *PSQI*, Pittsburgh Sleep Quality Index; *PHQ-9*, Patient Health Questionnaire-9; *GAD-7*, Generalized Anxiety Disorder Screener-7; *NRS*, numerical rating scale

*p value< 0.05 (Mann-Whitney test)

**p value< 0.01 (Mann-Whitney test)

^#^*p* value< 0.05 (Fisher’s exact test)

### Acute postoperative pain at movement during 24 h after surgery

**Fig. 1:**
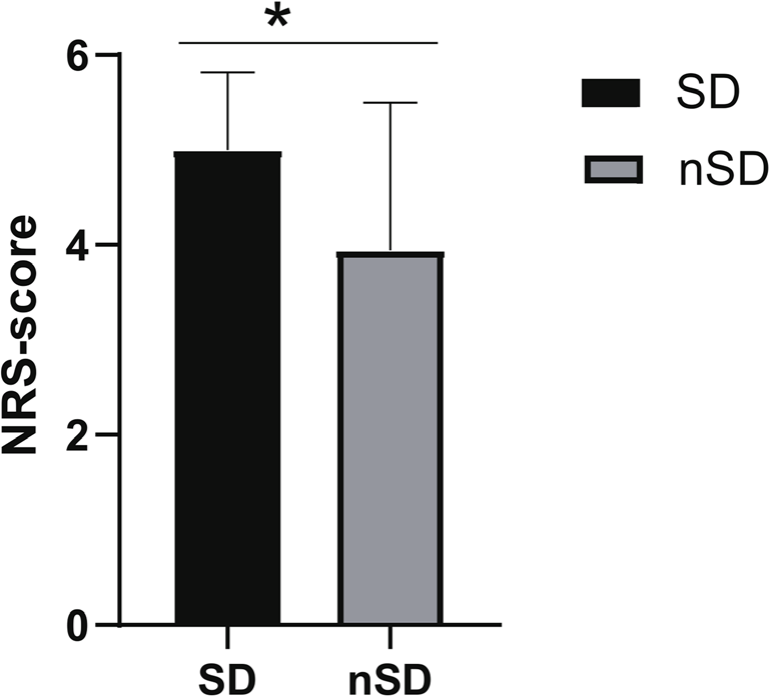
Women in the SD and nSD group showed significantly different pain experiences. The mean score of peak pain at movement during the 24 h after surgery was significantly higher in the SD group (5.00 ± 1.87, *p* = 0.019), while that of the nSD group was (3.94 ± 0.38). SD sleep disturbance (*n* = 19), nSD no sleep disturbance (*n* = 17)

The mean score of peak pain at movement during the 24 h after surgery was significantly higher in SD group (5.00 ± 1.87, *p* = 0.019), compared to nSD group (3.94 ± 0.38). More women in SD group (47%) asked for additional analgesic medication compared to nSD group (*p* = 0.03) (Fig. 1).

### Characteristics and alterations of the gut microbiota

**Fig. 2:**
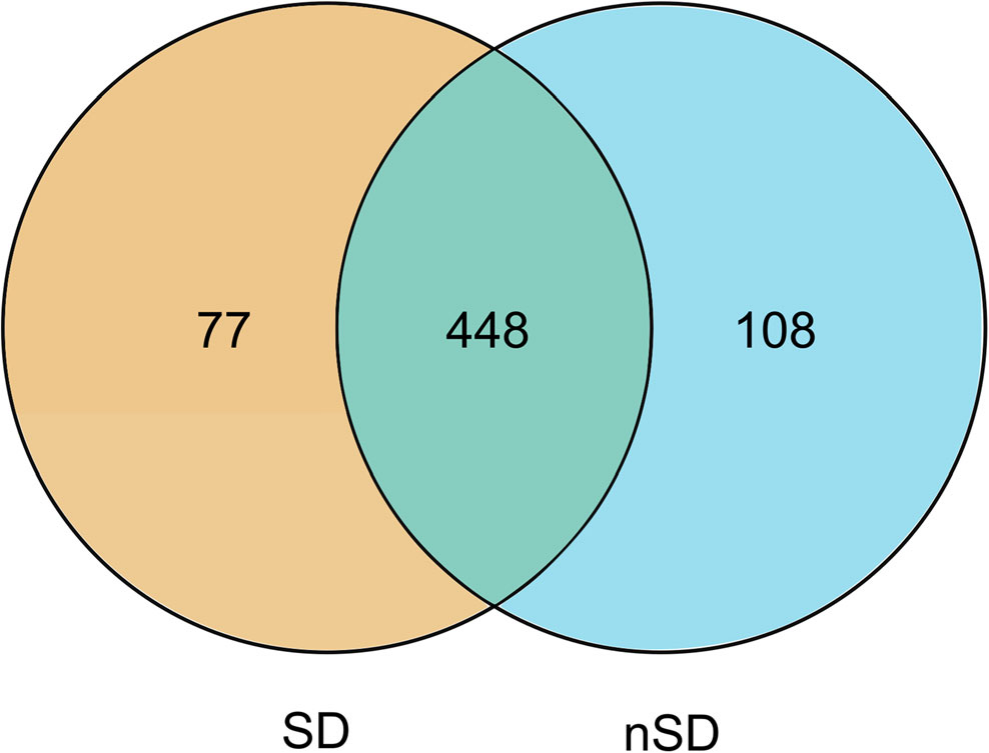
Veen diagram of the unique and shared OTUs between two groups. Fecal microbiota richness was significantly decreased in the SD group. OTU operational taxonomic unit, SD sleep disturbance (*n* = 19), nSD no sleep disturbance (*n* = 17)

**Fig. 3:**
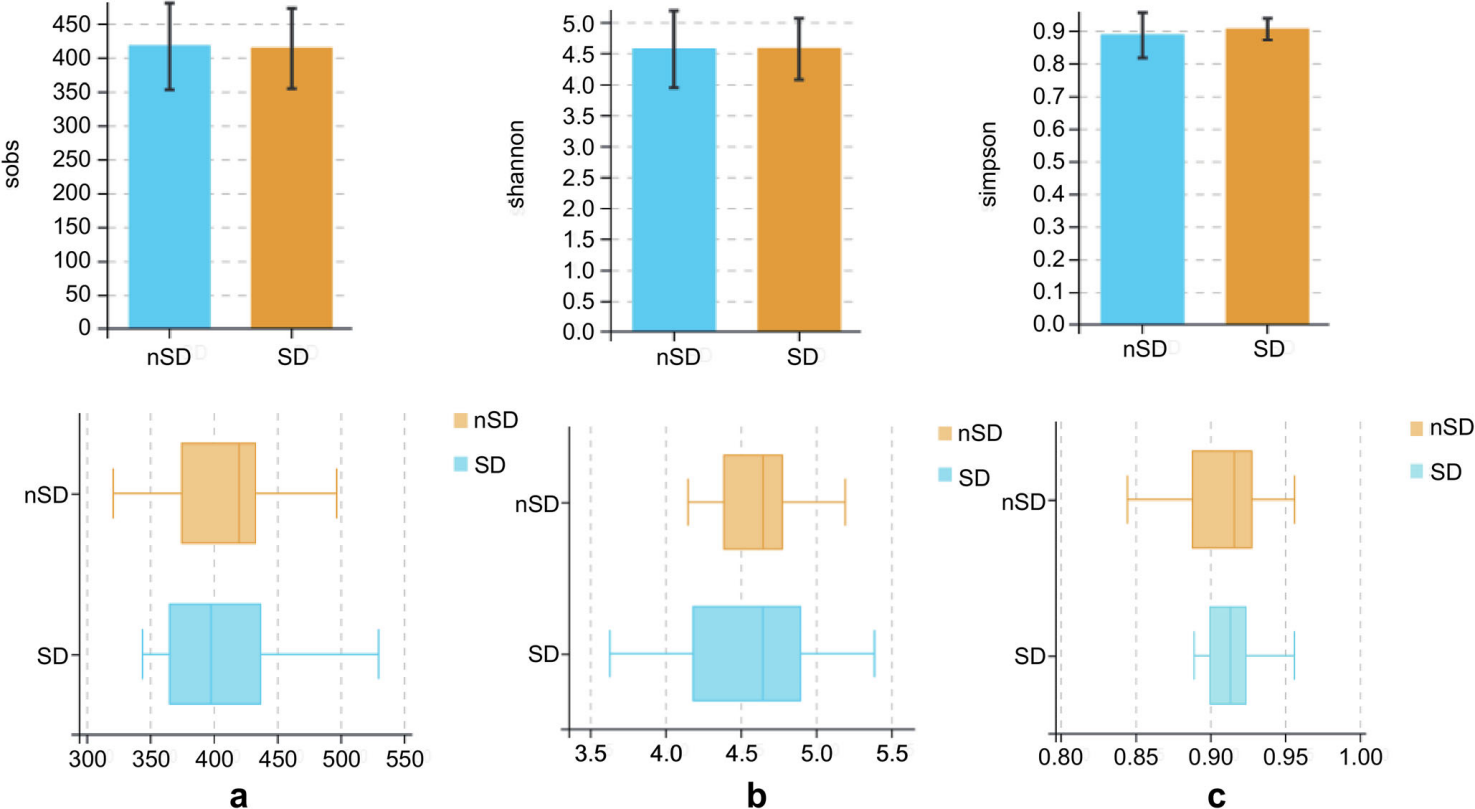
No difference in α-diversity between the two groups estimated by Sobs index, Shannon index, and Simpson index (*p* = 0.883, *p* = 0.997, *p* = 0.331, respectively). SD sleep disturbance (*n* = 19), nSD no sleep disturbance (*n* = 17)

**Fig. 4:**
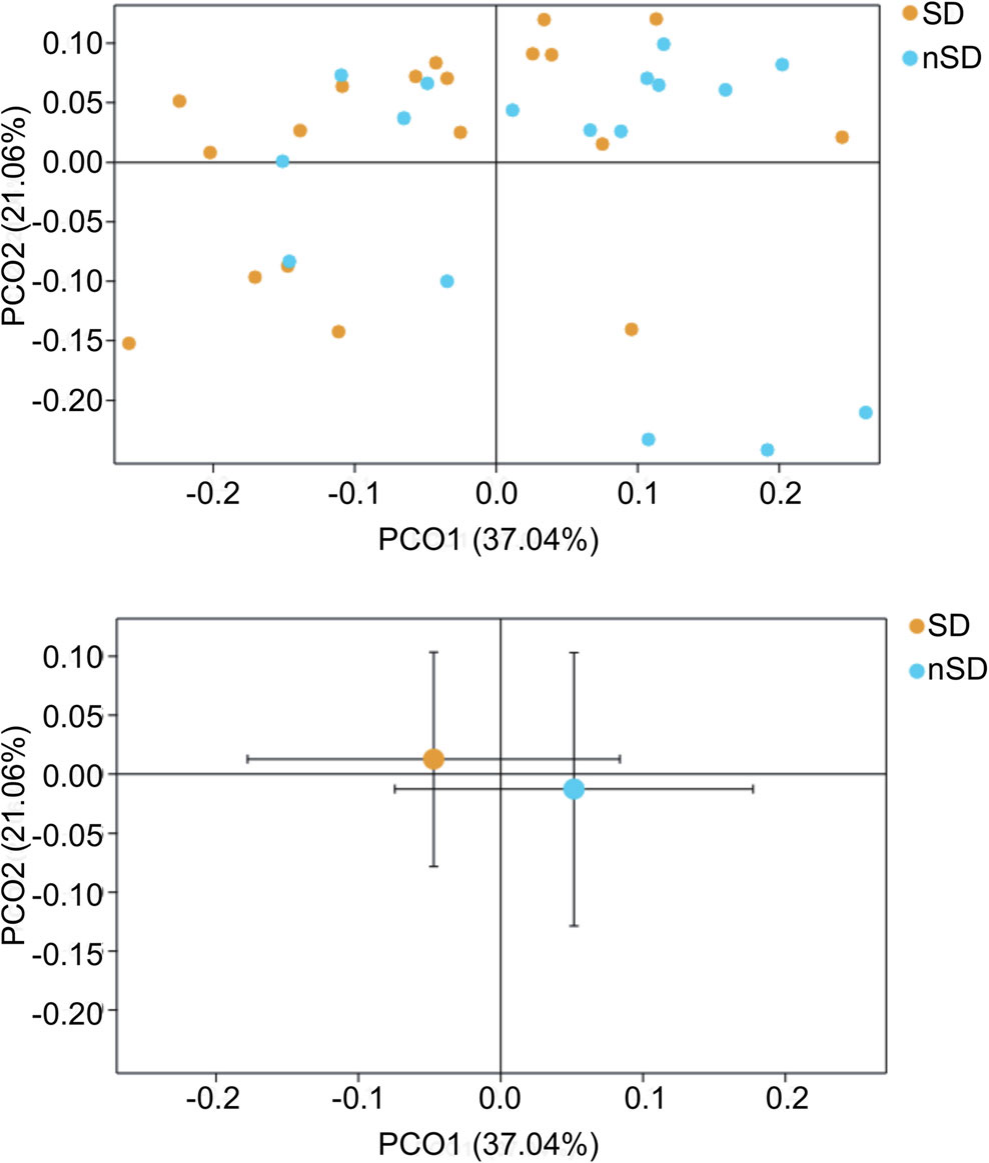
Beta diversity was calculated using weighted UniFrac by PCoA, indicating a symmetrical distribution of fecal microbial community among all the samples. Global community was significantly different using PERMANOVA (*p* = 0.02). PCoA Principal coordinate analysis, SD sleep disturbance (*n* = 19), nSD no sleep disturbance (*n* = 17)

**Fig. 5:**
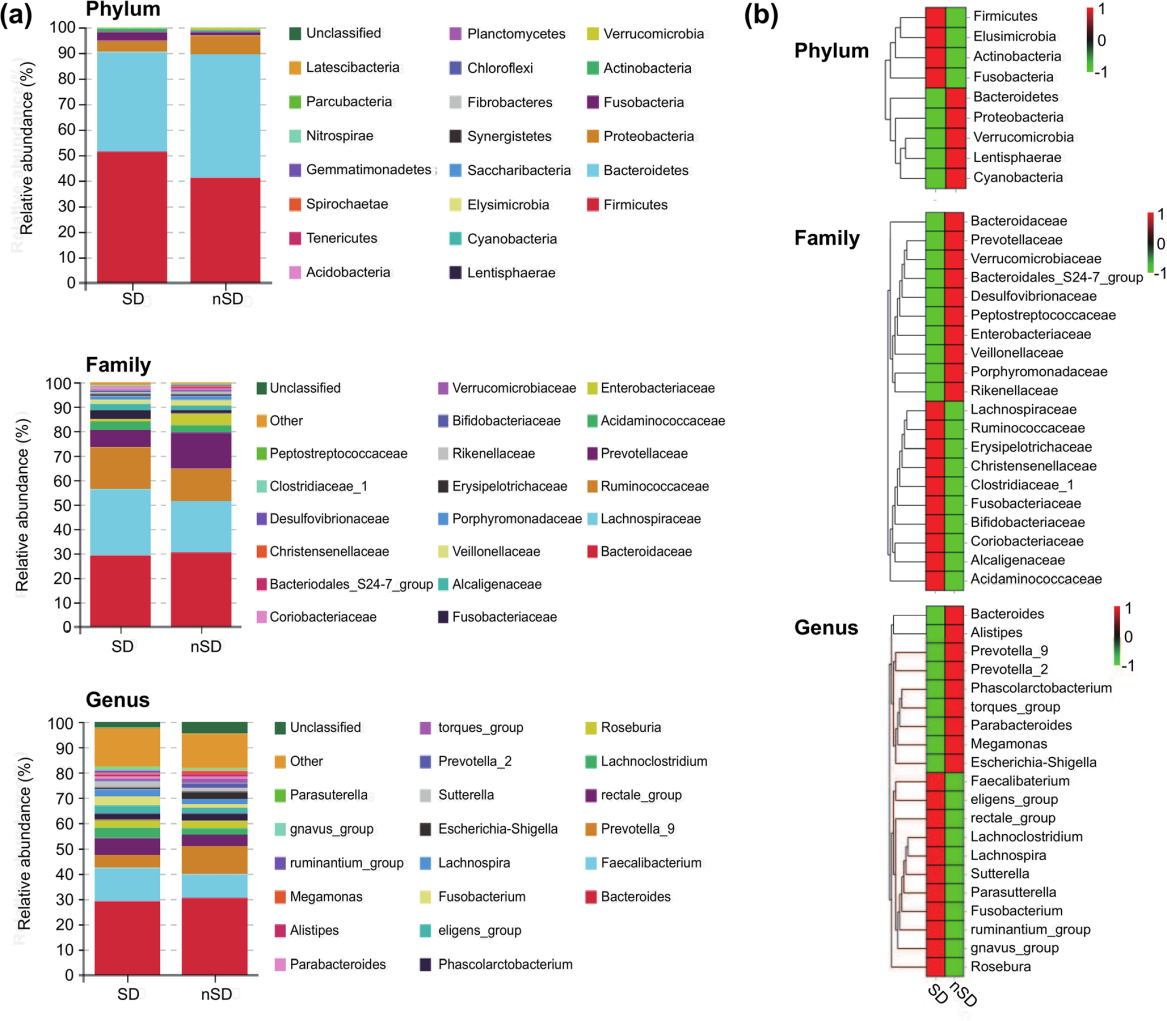
Distribution of intestinal microbiota between two groups. **a** Microbial composition at different levels. **b** Heat map of microbial relative abundance. SD sleep disturbance (*n* = 19), nSD no sleep disturbance (*n* = 17)

**Fig. 6:**
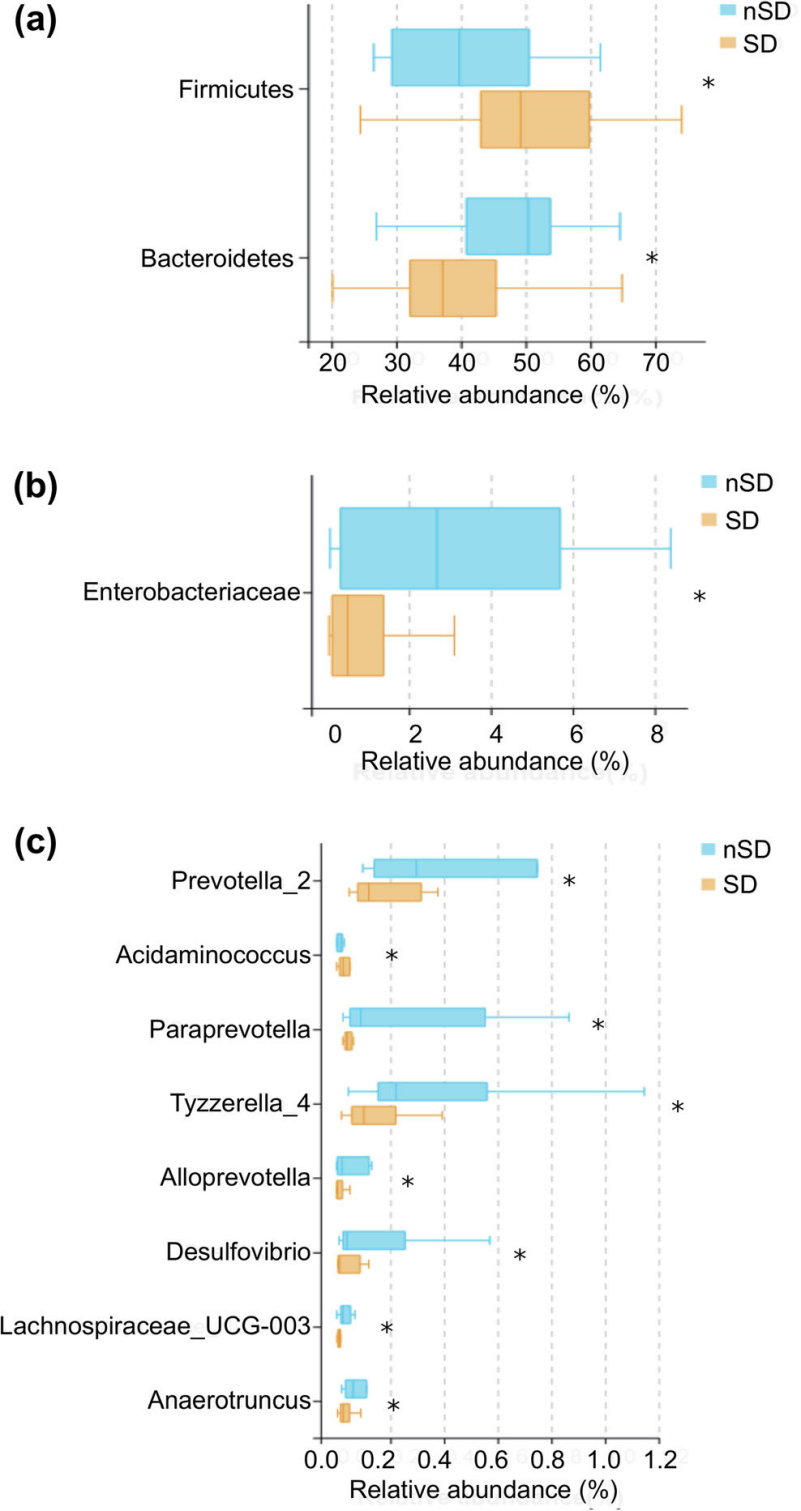
Significantly different intestinal microbiota between the two groups. **a** At the phylum level, the SD group had a higher relative abundance of Firmicutes and a lower relative abundance of Bacteroidetes. **b** At the family level, the SD group had a lower relative abundance of Enterobacteriaceae. **c** At the genus level, the SD group had a higher relative abundance of Acidaminococcus and a lower relative abundance of Alloprevotella, Desulfovibrio, Lachnospiraceae_UCG-003, Paraprevotella, Anaerotruncus, Prevotella_2, and Tyzzerella_4. SD sleep disturbance (*n* = 19), nSD no sleep disturbance (*n* = 17), *p < 0.05 (Wilcoxon rank sum test)

**Fig. 7:**
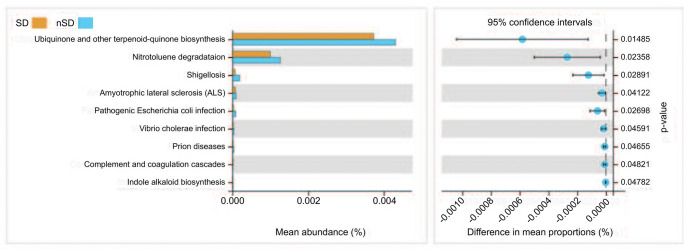
Heat map of different functions between two groups

Rarefaction curve analysis indicated that all samples reached a stable plateau, which meant the sampling was sufficient for most of the bacterial communities (Additional file 4). Fecal microbiota richness was decreased as the sleep quality declined (Fig. 2). There was no difference in α-diversity between the two groups, which was estimated by Shannon index, Simpson index, and Sobs index (Fig. 3). Principal coordinate analysis (PCoA) based on operational taxonomic unit (OTU) abundances reflected the β-diversities of the two groups. Using PERMANOVA, the calculated β-diversity indicated a significant difference between two groups (*p* = 0.02) (Fig. 4). The distribution of intestinal microbiota at different levels was also significantly different (Fig. 5). At the phylum level, the SD group had a higher relative abundance of Firmicutes (*p* = 0.021) and a lower relative abundance of Bacteroidetes (*p* = 0.013) compared to the nSD group, which resulted in higher F/B ratio in the SD group (Fig. 6a). At the family level, Enterobacteriaceae was much higher in the nSD group (*p* = 0.028) (Fig. 6b). At the genus level, the SD group harbored higher relative abundance of Acidaminococcus and lower relative abundance of Alloprevotella, Desulfovibrio, Lachnospiraceae_UCG-003, Paraprevotella, Anaerotruncus, Prevotella_2, and Tyzzerella_4 (Fig. 6c). We also observed significant differences in a few functions such as the ubiquinone and other terpenoid-quinone biosynthesis, the nitrotoluene degradation, and Shigellosis et al. (Fig. 7).

### Relationships between acute postoperative pain, clinical indicators, and gut microbiota

Genera Alloprevotella was negatively associated with peak pain at movement during the first 24 h (*r* = − 0.592, *p* < 0.001). The genus Desulfovibrio was negatively associated with symptoms of anxiety (*r* = − 0.451, *p* = 0.006), and genus Acidaminococcus was positively associated with white blood cell count (*r* = 0.346, *p* = 0.038). Partial correlations were performed to assess the relationships between sleep, acute pain, and the genus Alloprevotella, which suggested that the relationship between Alloprevotella and peak pain at movement during the first 24 h was not statistically significant after controlling for sleep (*r* = − 0.134, *p* = 0.443).

## Discussion

This preliminary study revealed an association between preoperative sleep disturbances, gut microbiota composition, and acute postoperative pain. Prevalence of preoperative sleep disturbance in our study is up to 53%, consistent with Fleming’s study of insomnia in breast cancer [5]. Women with preoperative sleep disturbances were significantly correlated with more severe peak pain at movement during 24 h after surgery, which is consistent with findings from previous studies [11, 12]. The gut microbiota was significantly different between women with and without sleep disturbance in this study, as other researchers have demonstrated [16, 17]. Women with sleep disturbance had a higher proportion of Firmicutes and a lower proportion of Bacteroidetes, resulting in higher F/B ratio that usually indicates gut microbiota dysbiosis [36].

There is an interesting trend toward significance of college education in the sleep disturbance group (*p* = 0.09). This finding conflicts with some previous studies in which a higher level of education was demonstrated to be associated with better sleep [37–40]. However, a similar trend has also been reported in studies of other diseases [41, 42]. Some possible explanations include (1) the small number of highly educated patients involved in this study, (2) the possibility that highly educated patients had taken more mental work with higher mental stress, such as worrying about career-limiting move or damaged appearance, and (3) habit of staying up late with brainwork which was confirmed by the subsequent visit.

The number of patients asking for additional analgesia after surgery was significantly higher in SD group compared to that of nSD group. According to this finding, we could adjust perioperative analgesic solutions for patients with sleep disturbance at the very beginning of their surgical decision, such as preemptive analgesia, a higher dose of operative and postoperative analgesia. Considering some side effects of traditional medication regimens including nausea and vomiting [43], other non-invasive with fewer adverse reactions but potentially effective methods are becoming a better choice, such as the intervention of gut microbiota which we proposed in the present study.

Alloprevotella, a genus that produces short-chain fatty acids, especially butyric acid, which can maintain the intestinal homeostasis [44], is negatively associated with peak pain at movement during 24 h after surgery. Alloprevotella was found as a benign bacterium [45], and its abundance negatively correlated with inflammation and type 2 diabetes mellitus [46, 47]. While previous studies have reported similar associations for different microbial strains, it is noteworthy that the body of evidence showing a relationship between changes to the gut microbiota and altered nociception [48], chemotherapy-induced mechanical hyperalgesia, and neuropathic pain is growing [49, 50].

Interestingly, the genus Desulfovibrio was negatively associated with preoperative symptoms of anxiety in the present study, and this disagrees with most published studies in which Desulfovibrio was regarded as an opportunistic pathogen and associated with diseases [51–53]. However, Desulfovibrio is a sulfate-reducing, anaerobic bacteria that is common in the colon. It is one of the most prominent producers of H_2_S, which is considered the third gasotransmitter and is involved in inflammation, gut motility, apoptosis, and many other vital biological functions [54]. Further studies are needed to develop the role of Desulfovibrio in sleep disturbance and pain.

Inflammation is associated with sleep quality and acute pain [55, 56]. The genus Acidaminococcus is positively associated with white blood cell count, which is regarded as an inflammatory biomarker together with neutrophil counts, platelet-lymphocyte ratio, and neutrophil-lymphocyte ratio. Acidaminococcus has been reported to be positively associated with state of diseases such as type 2 diabetes mellitus and obesity [57, 58].

In the present findings, the relationship between gut microbiota and postoperative pain became statistically non-significant after accounting for preoperative sleep disturbances, suggesting that preoperative sleep disturbances might result in both altered gut microbiota composition and more severe postoperative pain in breast cancer patients. Considered alongside previous studies, the present findings suggest altered gut microbiota composition as a possible link between preoperative sleep disturbances and increased acute postoperative pain. Evidence from healthy young adults suggests that a brief period of sleep restriction is sufficient to alter the composition of the gut microbiota and that altered microbiota plays a role in the processing of pain [16, 48]. As cross-sectional assessments can distort intermedial relationships [59], prospective studies are needed to explore whether or not sleep disturbance precedes the changes to the microbiota composition, which subsequently aggravate postoperative pain. Such studies could inform guidelines regarding probiotics and their potential role in improving intestinal health [60], creating a buffer against disturbed sleep-related acute pain.

The present findings must be considered in light of several study limitations. Although our study provides a wealth of information, the observational nature of the present study precludes any conclusions regarding causality. Using objective sleep quality measures (e.g., actigraphy) in future studies may provide more extensive and accurate information than that provided by the PSQI alone. Further studies in larger samples are also necessary to find the most relevant taxonomic rank for the relationships of interest (e.g., genus vs. species, among others). Other inflammatory biomarkers could be adopted, such as interleukin-6, interleukin-1 receptor antagonist, and tumor necrosis factor-α. Finally, the possible mechanisms underlying the relationships between sleep, acute pain, and gut microbiota composition, such as immunoregulation, were not examined in this study with the exception of the serum inflammatory state. Future studies should examine these mechanisms. This preliminary study provided evidence of a relationship between the gut microbiota composition, preoperative sleep quality, and acute postoperative pain in breast cancer patients undergoing elective surgery. These findings indicate a potential microbial pathomechanism in sleep-pain interactions thereby providing evidence for a practical and convenient preoperative intervention. Such an intervention may improve preoperative sleep quality and reduce acute postoperative pain as well as related complications in breast cancer patients, leading to improved effectiveness of rehabilitation and quality of life.

## Supplementary Information

ESM 1 (DOCX 11 kb)

ESM 2 (DOCX 11 kb)

ESM 3 (DOCX 13 kb)

ESM 4 (PNG 428 kb)

High resolution image (EPS 1451 kb)

## Data Availability

The data and materials are available from the corresponding author on reasonable request. The raw reads of 16S rRNA sequencing were deposited into the NCBI Sequence Read Archive (SRA) database (Accession Number: PRJNA566060).

## Abbreviations

BMI: body mass index
NRS: numerical rating scale
PSQI: Pittsburgh Sleep Quality Index
PHQ-9: Patient Health Questionnaire-9
GAD-7: Generalized Anxiety Disorder Screener-7
