# Supplementary material for: Relationships of sleep disturbance, intestinal microbiota, and postoperative pain in breast cancer patients: a prospective observational study
Source: Sleep Breath. 2020 Nov 19;25(3):1655–64. doi: 10.1007/s11325-020-02246-3 (PMC8376716; doi:10.1007/s11325-020-02246-3)
Supplement: Supplementary file 1 — (DOCX 11 kb) [file 11325_2020_2246_MOESM1_ESM.docx]

Inclusion criteria：

（1）breast cancer patients planning to undergo surgery

（2）women aged 18-65（including 18 and 65 years old）

（3）informed consent and voluntary participation in this clinical study

Exclusion criteria：

（1）use of antibiotics in the last one month

（2）history of mental or psychological problems, or are taking psychiatric medication or receiving professional psychotherapy

（3）history of cancer

（4）intestinal dysfunction (such as irritable Bowel Syndrome)

（5）metabolic diseases that affect the microbiota (e.g., thyroid dysfunction, diabetes, etc.)

（6）language communication difficulties
