# Supplementary material for: Relationships of sleep disturbance, intestinal microbiota, and postoperative pain in breast cancer patients: a prospective observational study
Source: Sleep Breath. 2020 Nov 19;25(3):1655–64. doi: 10.1007/s11325-020-02246-3 (PMC8376716; doi:10.1007/s11325-020-02246-3)
Supplement: Supplementary file 2 — (DOCX 11 kb) [file 11325_2020_2246_MOESM2_ESM.docx]

Standard Anesthesia Management

Standard anesthesia were performed by the same group of anesthesiologists who were familiar with the process and trained in advance. Target-controlled infusion (TCI) of propofol-remifentanil was used throughout the anesthesia, with the target effect-site concentration of propofol between 3-6 ug/ml and that of remifentanil between 4-8ng/ml. Rocuronium was used with a dosage of 0.3mg/kg for intubation. After anesthesia induction, 2-2.5% sevoflurane was used in combination with propofol and remifentanil to maintain proper anesthetic depth and avoid intraoperative awareness. Vasoactive drugs (such as ephedrine, urapidil) were used when necessary. No other sedatives were used during whole anesthesia. 40mg parecoxib sodium was given 5min before skin incision. Sufentanil was given with a dosage of 0.2ug/kg 30 min before the end of the surgery. Intraoperative fluids were crystalloid except in emergency circumstances. All patients were transferred to Postanesthesia Care Unit (PACU) for further monitor after surgery until they achieved the safty criteria of return to the ward.
