# Supplementary material for: Relationships of sleep disturbance, intestinal microbiota, and postoperative pain in breast cancer patients: a prospective observational study
Source: Sleep Breath. 2020 Nov 19;25(3):1655–64. doi: 10.1007/s11325-020-02246-3 (PMC8376716; doi:10.1007/s11325-020-02246-3)
Supplement: Supplementary file 3 — (DOCX 13 kb) [file 11325_2020_2246_MOESM3_ESM.docx]

1. **DNA extraction and PCR amplification**

Microbial DNA was extracted using the HiPure Soil DNA Kits (or HiPure Stool DNA Kits) (Magen, Guangzhou, China) according to manufacturer’s protocols. The 16s rDNA V3-V4 region of the ribosomal RNA gene were amplified by PCR（95 ℃ for 2 min, followed by 27 cycles at 98℃ for 10 s, 62 ℃ for 30 s, and 68 ℃ for 30 s and a final extension at 68℃ for 10 min）using primers 341F: CCTACGGGNGGCWGCAG; 806R: GGACTACHVGGGTATCTAAT, where the barcode is an eight-base sequence unique to each sample. PCR reactions were performed in triplicate 50μL mixture containing 5μL of 10 × KOD Buffer, 5μL of 2.5 mM dNTPs, 1.5μL of each primer (5 μM), 1μL of KOD Polymerase, and 100 ng of template DNA.

1. **Illumina Hiseq 2500 sequencing**

Amplicons were extracted from 2% agarose gels and purified using the AxyPrep DNA Gel

Extraction Kit (Axygen Biosciences, Union City, CA, U.S.) according to the manufacturer’s

instructions and quantified using ABI StepOnePlus Real-Time PCR System (Life Technologies，

Foster City, USA). Purified amplicons were pooled in equimolar and paired-end sequenced (2 ×

250) on an Illumina platform according to the standard protocols.

**3. Quality control and reads assembly**

**3.1. Reads filtering**

Raw data containing adapters or low quality reads would affect the following assembly and analysis. To get high quality clean reads, raw reads were further filtered according to the following rulesusing FASTP (https://github.com/OpenGene/fastp): 1) Removing reads containing more than 10% of unknown nucleotides (N); 2) Removing reads containing less than 80% of bases with quality (Q-value)＞20.

**3.2. Reads assembly**

Paired end clean reads were merged as raw tags using FLSAH (version 1.2.11) with a minimum overlap of 10bp and mismatch error rates of 2%.

**3.3. Raw tag filtering**

Noisy sequences of raw tags were filtered by QIIME (version 1.9.1 ) pipeline under specific filtering conditions to obtain the high-quality clean tags.

**3.4. Chimera checking and removal**

Clean tags were searched against the reference database (http://drive5.com/uchime/uchime_download.html) to perform Reference-based chimera checking using UCHIME algorithm (http://www.drive5.com/usearch/manual/uchime_algo.html). All chimeric tags were removed and finally obtained effective tags were used for further analysis.
