# Supplementary figures and images for: Relationships of sleep disturbance, intestinal microbiota, and postoperative pain in breast cancer patients: a prospective observational study
Source: Sleep Breath. 2020 Nov 19;25(3):1655–64. doi: 10.1007/s11325-020-02246-3 (PMC8376716; doi:10.1007/s11325-020-02246-3)

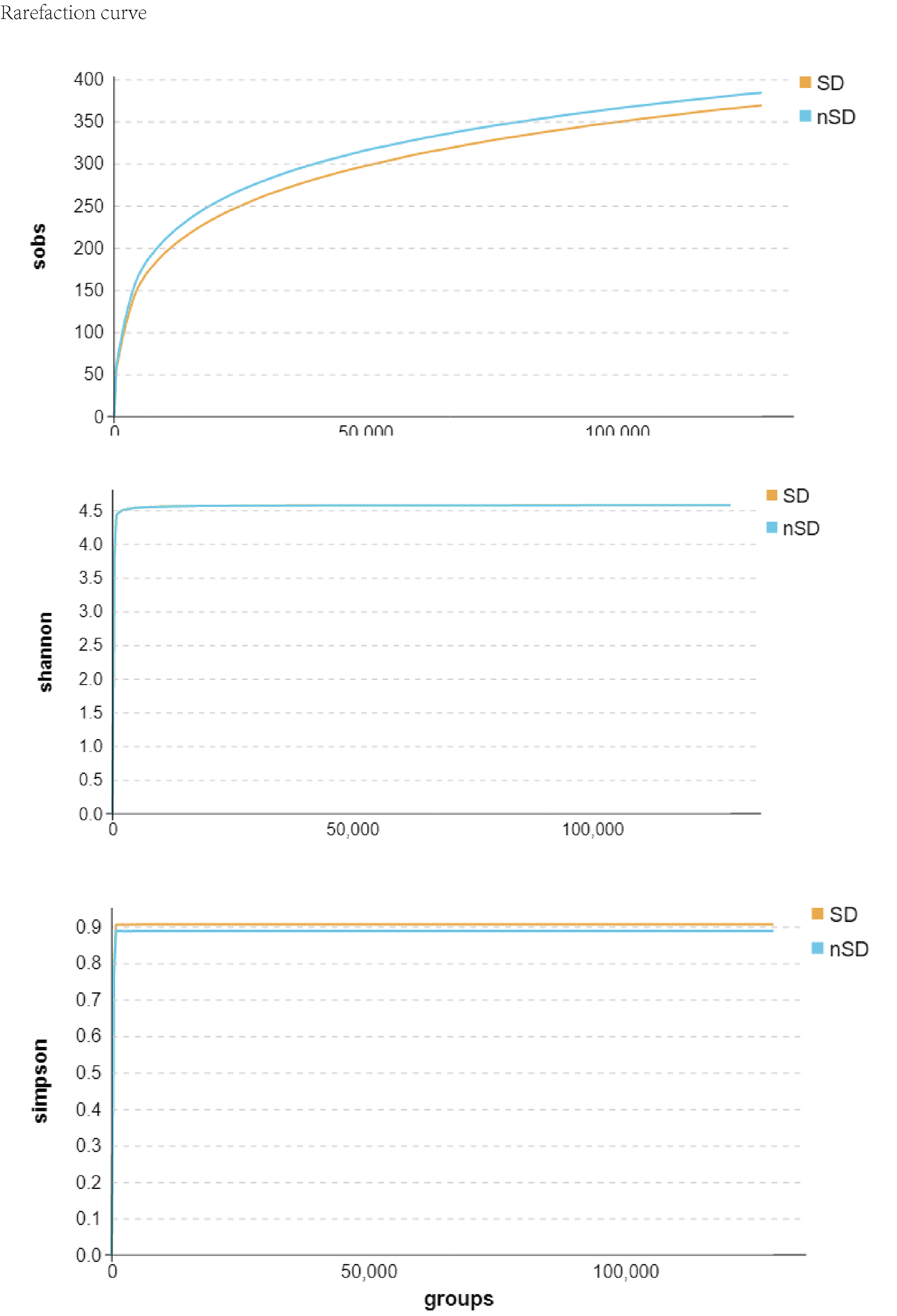

Supplement: Supplementary file 4 — (PNG 428 kb) [file 11325_2020_2246_Fig8_ESM.png]
